# Chicago Academy of Medical Sciences

**Published:** 1861-02

**Authors:** E. L. Holmes

**Affiliations:** Secretary; Chicago


### CHICAGO ACADEMY OF MEDICAL SCIENCES.

CHICAGO, *February* 1*st*, 1861.

The regular monthly meeting of the Academy was held at its rooms this evening, the President, Dr. Bloodgood, in the chair. After the reading of the minutes of the last meeting, the President appointed the following committee in accordance with Art. VI, Sec. II (amended) of the Constitution:

*Anatomy and Physiology*—Dr. E. Powell.
*Theory and Practice*—Dr. A. Fisher.
*Obstetrics and Diseases of Women*—Dr. O. Smith.
*Chemistry and Materia Medica*—Dr. J. H. Rauch.
*Public Health*—Prof. N. S. Davis, M. D.
*Surgery*—Prof. J. W. Freer, M. D.
*Pathology and Histology*—Prof. J. H. Hollister, M. D.

Dr. R. C. Hamill read an interesting report of a case of inversio uteri. The labor was conducted with little difficulty, although there was a slight antero-posterior contraction of the pelvis. During the last couple of hours previous to the delivery of the child, five or six drops of chloroform were administered from time to time, on account of nervous excitement. Ten or fifteen minutes after delivery, the uterus being well contracted, a violent pain seized the patient, when the placenta was suddenly expelled. Alarming prostration and flooding followed. On examination, the uterus was found inverted. Fortunately, after several attempts, the organ was replaced. Although there were subsequent symptoms of metritis, they were relieved by quinine, veratrum viride and hyoscyamus, and the patient soon recovered perfect health. It should be observed that no force was applied to the cord in the delivery of the placenta.

Dr. H. N. Hurlbut reported orally a case in which he was recently called to a patient, married, and about 40 years of age, suffering from violent hemorrhage and prostration. On examination, the uterus was found inverted and presenting evident post partum appearances. The organ was readily reduced and the patient recovered without unfavorable symptoms. No child or placenta was any where observed, and no satisfactory account of the case could be obtained from the family.

Dr. Bevan related the case of a woman, aged about 35 years, in the 5th or 6th month of pregnancy, who stated that after injury in a quarrel, followed by violent exercise, the usual motions of the fœtus suddenly ceased. No fœtal pulsations could be heard and no motions could be induced either by pressure or by the application of cold to the abdomen. In three weeks the woman was delivered of a fœtus in a state of decomposition. No blood passed from the uterus and vagina, except in the form of dark, decomposed clots.

The subject of Pneumonia, assigned at the last meeting, was discussed at some length by Drs. O. Smith, Johnson, Heydock, Fisher, Davis, Wickersham, Bevan, Hamill, Andrews, McAllister and Bloodgood.

Subject of discussion at the next meeting—The Diagnosis of Cerebral Diseases of Infancy.

Adjourned.

E. L. HOLMES, Secretary.

